# End-to-End Prediction of Chloride Concentration Profiles in Marine Concrete Using Machine Learning

**DOI:** 10.3390/ma19214509

**Published:** 2026-10-26

**Authors:** Chao Wang, Chaoyu Liu, Weiyang Wang, Yu Zhang

**Affiliations:** 1 Marine and Floating Engineering Technology Center, Mingyang Smart Energy Group Co., Ltd., Zhongshan 528437, China; wangchao01@mywind.com.cn; 2 Key Laboratory of Concrete and Prestressed Concrete Structures of the Ministry of Education, Southeast University, Nanjing 210096, China; liuchaoyu59@gmail.com (C.L.); 101013462@seu.edu.cn (Y.Z.)

**Keywords:** chloride concentration profile, end-to-end prediction, machine learning, marine concrete

## Abstract

For durability design of concrete structures exposed to marine environments, the relationship between chloride concentration and penetration depth is critical as it determines the theoretical onset of rebar corrosion and thickness of the protective layer. However, current codes across the world typically rely on deterministic environmental category and design service life of concrete structure to obtain the value of thickness. To address this issue, the authors proposed an end-to-end machine learning method that directly predicted chloride concentration profiles from input variables, without estimating intermediate parameters, e.g., surface chloride concentration and chloride diffusion coefficient. Field exposure data was collected and compiled from 63 published studies, covering 44 exposure sites across 16 countries, yielding a total of 6365 data samples representing 701 chloride concentration profiles. Six end-to-end machine learning models were developed, among which tabular prior-data fitted network (TabPFN) performed best within the constructed dataset, achieving a 0.858 ± 0.025 for testing *R*^2^. SHapley Additive exPlanations (SHAP) analysis identified exposure depth and exposure duration as the dominant input features, and the spatial and temporal evolution of chloride concentration formed the principal basis for model prediction. Through parametric investigation and external validation, it was found that the TabPFN model can reasonably describe the attenuation of chloride concentration with depth and effectively capture the influences of different factors on chloride diffusion within concrete. With the designed GUI, the authors believed that it can be a convenient tool for the rapid prediction of chloride concentration, providing useful insights for subsequent durability assessment.

## 1. Introduction

For concrete structures exposed to marine environments, chloride ions penetrate into the concrete and accumulate on the surface of reinforcement, serving as the primary initiation factor of steel corrosion [1]. Corrosion would lead to a reduction in the cross-sectional area of reinforcing bar, a decline in the bonding strength between concrete and steel, cracking/spalling of the protective layer, and thus a decrease in the bearing capacity of concrete members and structures [2].

For durability design, the relationship between chloride concentration and penetration depth is critical as it determines the theoretical onset of rebar corrosion and thickness of the concrete protective layer. However, current design codes worldwide typically determine the concrete cover thickness based on deterministic environmental exposure categories and the design service life of concrete structures [3], which is challenging to fully account for factors, e.g., the time-varying nature of chloride transport, uncertainties in material and environmental variables, etc. Therefore, a model that can accurately predict the spatiotemporal distribution of chloride concentration in concrete is essential, which can provide a robust foundation for durability design and reliability analysis.

This study on chloride transport in concrete is predominantly based on Fick’s second law of diffusion [4]. Given assumptions of a homogeneous semi-infinite medium, a constant surface chloride concentration, and a constant apparent diffusion coefficient, the model can describe the variation of chloride concentration with exposure duration and penetration depth through an error function solution. However, these assumptions cannot always be satisfied in the actual environment. For example, Mangat and Molloy [5] found that the chloride diffusion coefficient exhibited a notable time dependence and thus introduced a time-varying diffusion coefficient to predict the long-term chloride concentration in concrete. With long-term field and laboratory exposure data, Thomas and Bamforth [6] investigated the influence of fly ash and ground granulated blast furnace slag (slag for short) on chloride diffusion in concrete and their time-dependent features. Further, Boddy et al. [7] proposed a chloride diffusion model that incorporated multiple transport processes and considered the time-varying characteristics of concrete properties. Nokken et al. [8] quantified the reduction of chloride diffusion coefficient with exposure time according to laboratory trials up to three years. Ju et al. [9] developed a chloride concentration profile prediction model applicable to marine tidal environment within the framework of Fick’s second law of diffusion, taking into account the water to cement ratio, the volume fraction and maximum particle size of coarse aggregate. However, note that these predictions remained highly dependent on, e.g., concrete compositions, aggregate characteristics, environmental features, etc., and laboratory experiments were mandatory to obtain some key parameters.

In recent years, machine learning methods have gradually been applied to predict chloride diffusion behavior in concrete, owing to their ability to extract multivariate features and perform nonlinear mappings. Among these studies, emphasis was primarily laid on estimating critical parameters of traditional diffusion models, especially for surface chloride concentration and chloride diffusion coefficient. Cai et al. [10] constructed an integrated machine learning model that employed concrete composition, experimental condition, and exposure duration as inputs to estimate surface chloride concentration of marine concretes. Ahmad et al. [11] assessed the applicability of, e.g., gene expression programming, artificial neural network (ANN), and decision tree in predicting surface chloride concentration of concretes containing supplementary cementitious materials (SCMs). On the other hand, Tran et al. [12] compared the prediction performance of eight algorithms, including support vector regression (SVR), extreme learning machine, k-nearest neighbor (KNN), etc., for chloride diffusion coefficient. Additionally, they used SHapley Additive exPlanations (SHAP) and individual conditional expectation to illustrate the impact of each input. The above research demonstrates that data-driven machine learning approaches can identify the complex nonlinear relationships among concrete composition [13], environmental condition, exposure duration, and chloride transport behavior in concrete. However, the outputs of these models were intermediate parameters, i.e., surface chloride concentration and chloride diffusion coefficient [10,11,12,14], limiting their direct application for durability design.

Therefore, a few investigations began to concentrate explicitly on the spatiotemporal chloride concentration distribution. Based on measurement data of 600 days’ exposure to a coastal environment, Wu et al. [15] estimated the prediction performance of different deep learning-based models for chloride concentration profile, which were composed of convolutional neural network (CNN), long short-term memory (LSTM), etc. These models performed differently, of which LSTM outperformed the others in terms of prediction accuracy. With 918 sets of experimental data, Aliasghar-Mamaghani et al. [16] utilized machine learning methods to quantitatively describe the spatiotemporal evolution of chloride content in concrete. The voting regressor combining various algorithms was found to be the most promising approach in reproducing chloride concentration. Nevertheless, studies on direct chloride concentration profile prediction remained limited. The generalization of these developed models across different concrete compositions, environmental conditions, as well as time scales, required further validation.

To this end, the authors of this paper proposed an end-to-end machine learning method to predict the chloride concentration profile in concrete and attempted to establish a mapping relationship among concrete composition, environmental condition, exposure duration, exposure zone/spatial location, and chloride concentration. This approach reduced error propagation caused by the incorporation of intermediate parameters and improved convenience of the model for engineering application. A comprehensive multi-source chloride concentration dataset was constructed first, followed by feature processing, hyperparameter optimization, and model training. Next, performance of six machine learning models was compared on both training and testing sets, and SHAP analysis was adopted to examine the model interpretability. In addition, the potential of the best performed model for durability design was explored with parametric investigation and external validation. Finally, a graphical user interface (GUI) was designed as a convenient tool for the rapid prediction of chloride concentration profile, providing useful insights for subsequent durability assessment.

## 2. Materials and Methods

### 2.1. Dataset Construction

#### 2.1.1. Data Sources

This paper considered both Ordinary Portland Cement (OPC) concrete and concrete incorporating SCMs, e.g., fly ash and slag, which were among those most used in concrete structures exposed to marine environments. Three representative exposure groups, i.e., tidal, splash, and submerged zones, were examined. Field exposure data was collected and compiled from 63 published studies, covering 44 exposure sites across 16 countries. Detailed information on the compiled database is provided in the Supplementary Materials. To the best of the authors’ knowledge, the yielded dataset constituted the most comprehensive and geographically extensive compilation of long-term natural exposure data available to date. It therefore provided a robust basis for developing machine learning models to predict chloride concentration profile in concrete under different environmental conditions.

#### 2.1.2. Data Preprocessing

The raw data collected from studies exhibited significant heterogeneity arising from variations in experimental procedure, unit definition, and reporting convention. To ensure data comparability and preserve the physical consistency required for model training, all data were systematically preprocessed to standardize the definition and unit of chloride concentration.

**Figure 1:**
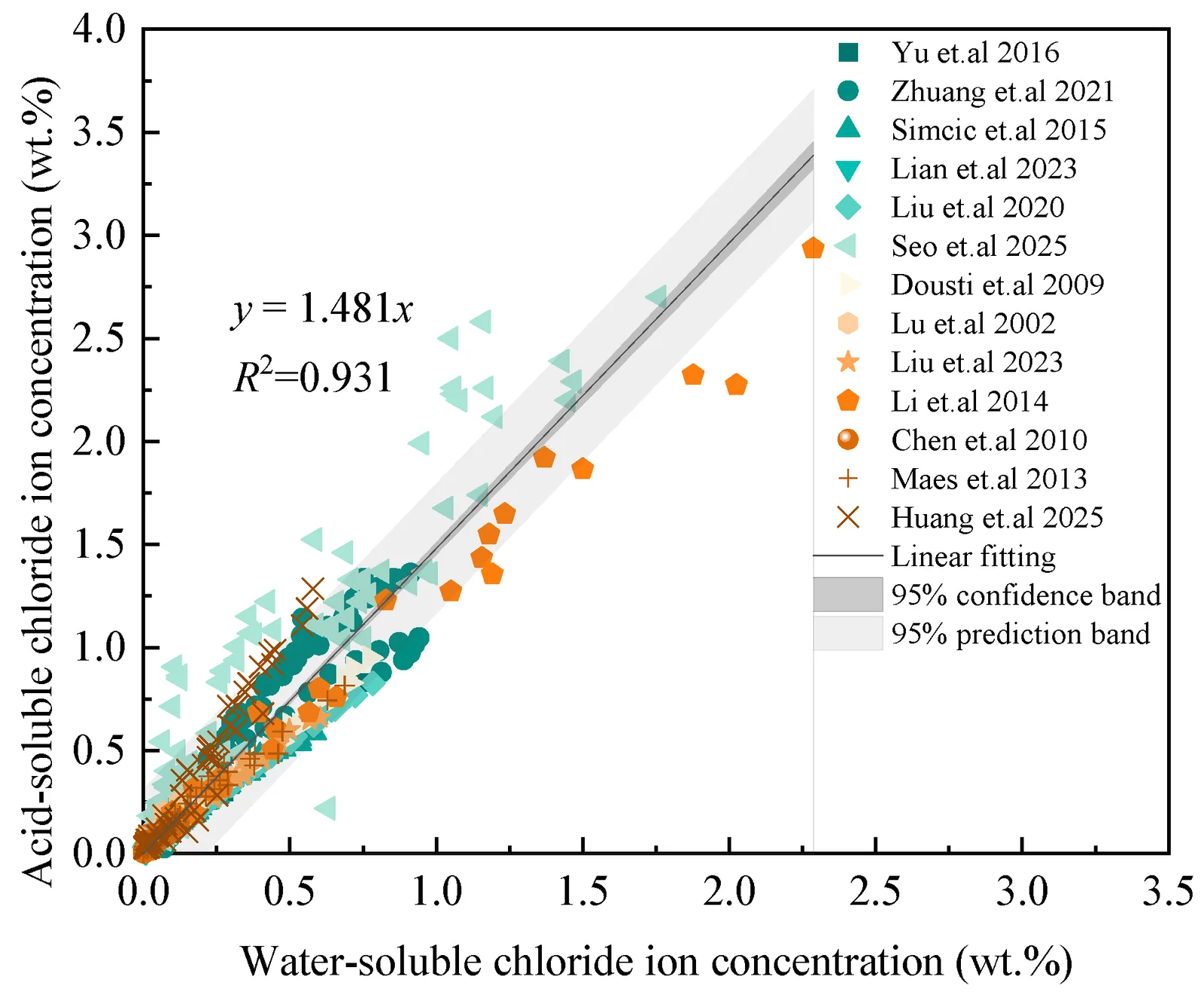
Relationship between water- and acid-soluble chloride concentrations [30]. Corresponding references are listed as follows: Yu et al. [17], Zhuang et al. [18], Simcic et al. [19], Lian et al. [20], Liu et al. [21], Seo et al. [22], Dousti et al. [23], Lu et al. [24], Liu et al. [25], Li et al. [26], Chen et al. [27], Maes et al. [29], Huang et al. [28].

The definition of chloride concentration was standardized first. Chloride concentration of concrete reported in the literature was generally obtained utilizing either water-soluble or acid-soluble extraction methods. As acid-soluble testing was mostly adopted in the studies, all reported chloride concentrations were converted to acid-soluble equivalents to minimize systematic discrepancy. Figure 1 summarizes the results from 13 published papers that provided both water-soluble and acid-soluble chloride concentrations for the same concrete specimens [17,18,19,20,21,22,23,24,25,26,27,28,29]. Despite measurement-related uncertainties across different binder systems and exposure conditions that may be introduced, a linear regression relationship between these two measurements can be established, as expressed in Equation (1). The yielded coefficient of determination (*R*^2^ = 0.931) indicated a strong correlation and a satisfactory goodness of fitting. Based on this empirical relationship, all water-soluble chloride concentrations were transformed into their corresponding acid-soluble equivalents.

*C_Cl_*_,*acid*_ = 1.481 ⋅ *C_Cl_*_,*water*_ (1)

where *C_Cl_*_,*acid*_ and *C_Cl_*_,*water*_ denote the acid- and water-soluble chloride concentrations, respectively.

Subsequently, the unit of chloride concentration was normalized. In this paper, all chloride concentrations were recalculated relative to the mass of concrete, using the reported mix proportion in the corresponding studies.

#### 2.1.3. Statistical Distribution and Feature Correlation

**Figure 2:**
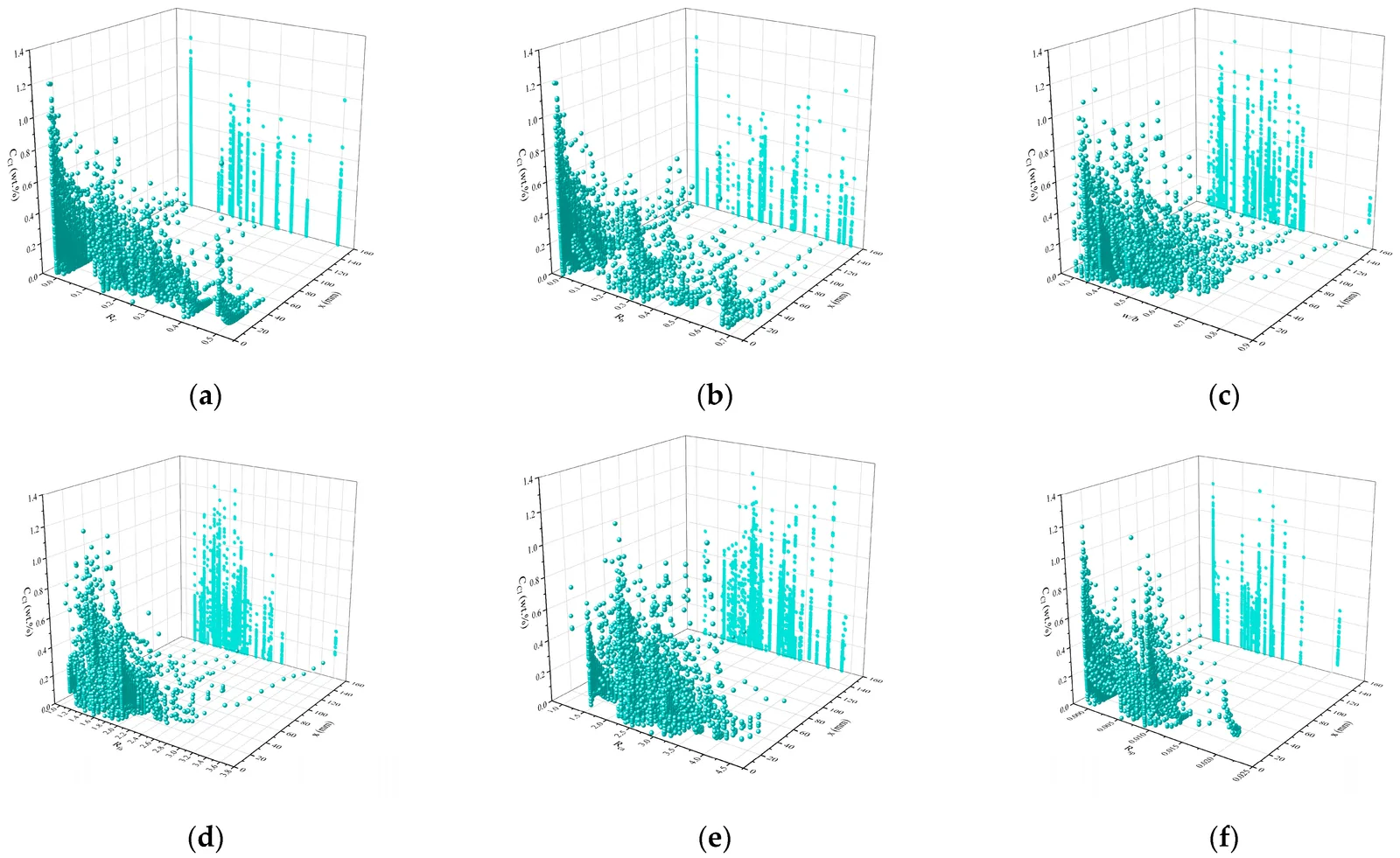

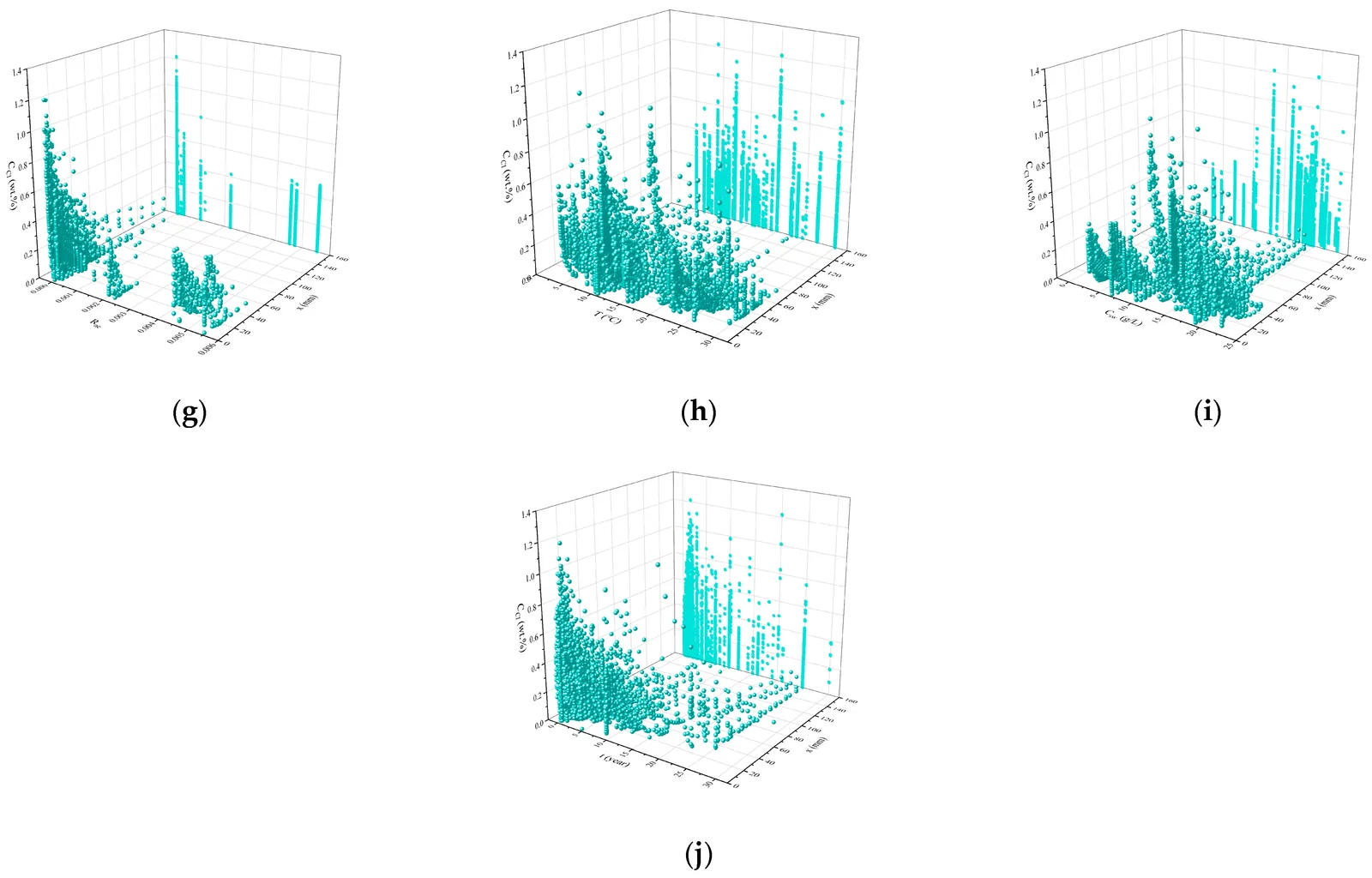
Three-dimensional scatter distribution of *C_Cl_* with respect to exposure depth and other feature variables: (**a**) *R_f_*-*x*-*C_Cl_*; (**b**) *R_b_*-*x*-*C_Cl_*; (**c**) *w/b*-*x*-*C_Cl_*; (**d**) R_fa_-x-C_Cl_; (**e**) R_ca_-x-C_Cl_; (**f**) R_sp_-x-C_Cl_; (**g**) R_ae_-x-C_Cl_; (**h**) T-x-C_Cl_; (**i**) C_sw_-x-C_Cl_; (**j**) *t*-*x*-*C_Cl_*.

Twelve input features were selected in the paper, including one categorical feature (exposure zone) and 11 numerical features: fly ash-to-binder ratio *R_f_*, slag-to-binder ratio *R_b_*, water-to-binder ratio *w*/*b*, fine aggregate-to-binder ratio *R_fa_*, coarse aggregate-to-binder ratio *R_ca_*, water reducer-to-binder ratio *R_sp_*, air entraining agent-to-binder ratio *R_ae_*, mean ambient temperature *T*, seawater chloride concentration *C_sw_*, exposure duration *t*, and exposure depth *x*. It should be mentioned that, due to the limitation of current dataset, missing values of seawater chloride concentration were imputed with the standard seawater salinity of 19.0 g/L, whereas the other numerical features were replaced with their respective medians. Among these features, the highest missing ratio was observed for *R_ae_* (21.70%), followed by *R_fa_* and *R_ca_* (15.11% and 14.45%). As a result, a total of 6365 data samples representing 701 chloride concentration profiles were generated. Among them, the splash, tidal, and submerged zones consisted of 858, 3694, and 1813 samples, corresponding to 115, 395, and 191 chloride concentration profiles, respectively. Statistical summaries of the 11 numerical features are given in Table 1, and their distributions are depicted in Figure 2. Note that the data were distributed unevenly among exposure zones (rich in the tidal region), and the subsequent dataset partitioning was thus stratified by exposure zone to maintain a consistent proportion of each zone in the training and testing sets.

**Figure 3:**
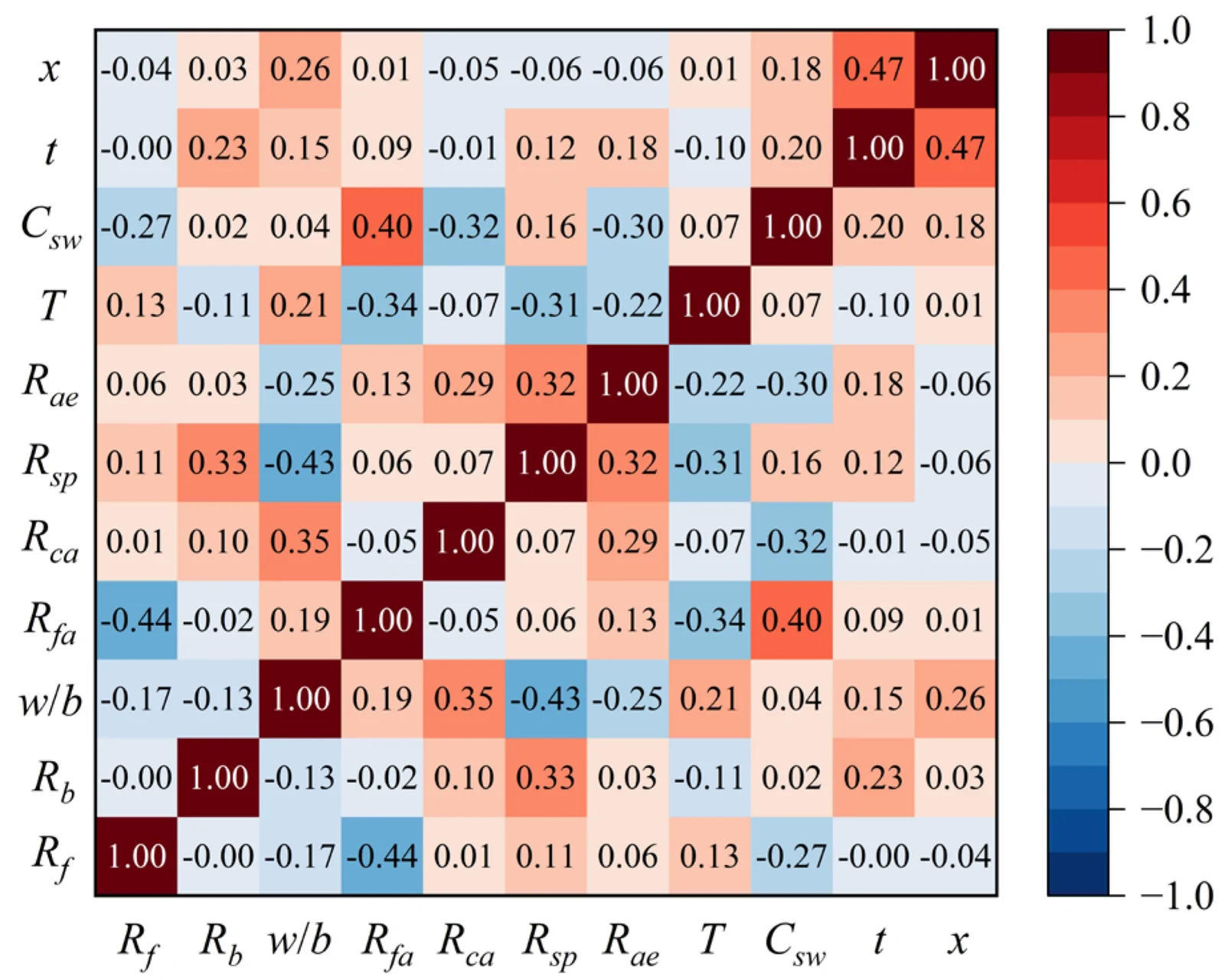
Pearson correlation heatmap of 11 numerical input features.

To evaluate the linear correlations among 11 numerical input features, a Pearson correlation heatmap is plotted in Figure 3. As can be seen, the strongest positive correlation was found between exposure duration and exposure depth, with a correlation coefficient of 0.47. On the other hand, fly ash and fine aggregate to binder ratios were mostly negatively correlated (−0.44). Overall, correlations among these features were weak, suggesting that the input variables characterized concrete composition and environmental conditions from different perspectives.

### 2.2. Machine Learning Methods

#### 2.2.1. Overview of Machine Learning Algorithms

**Figure 4:**
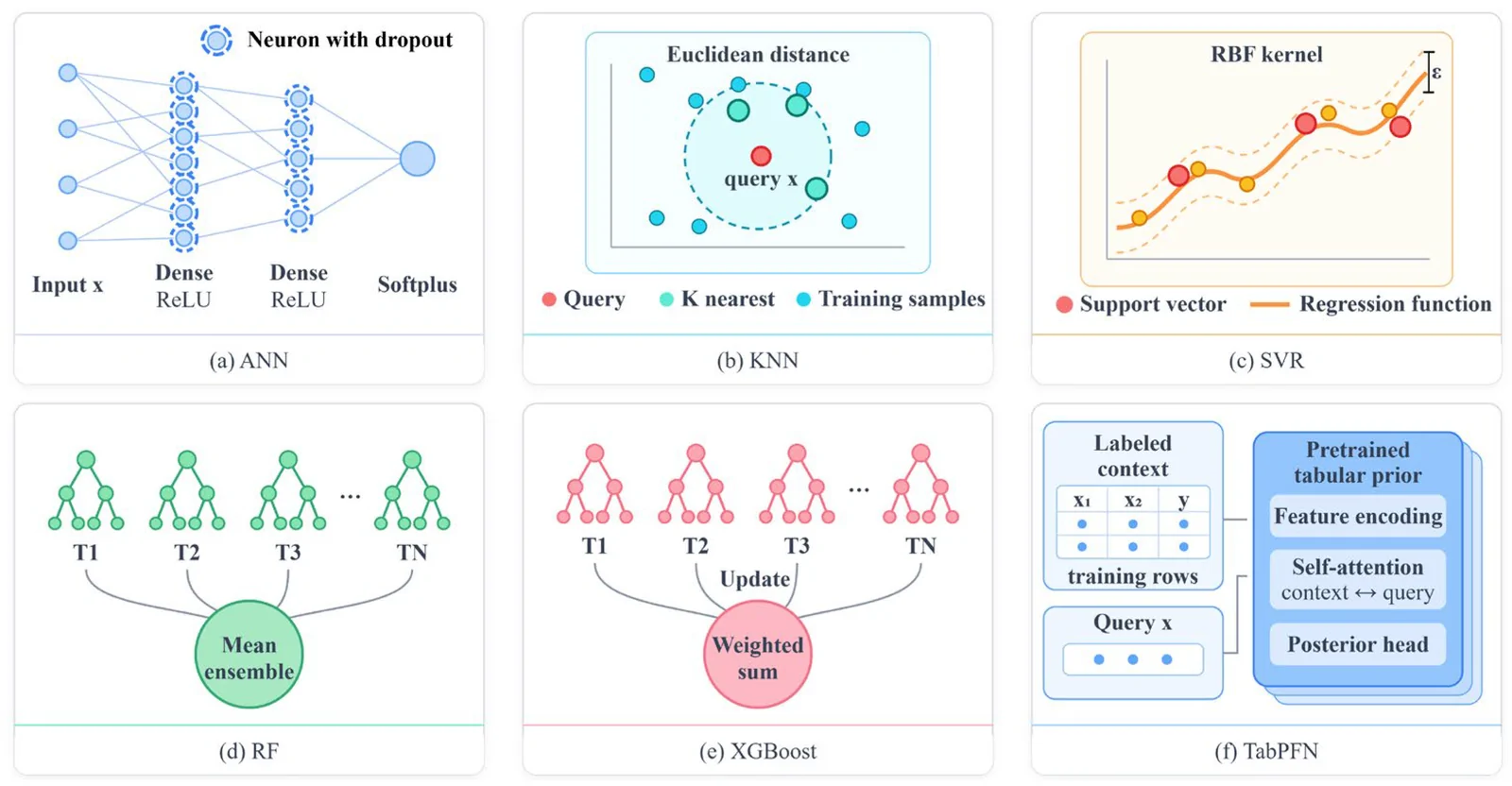
Architectures of six machine learning models.

Six models were put forward in the paper, i.e., ANN, KNN, SVR, random forest (RF), extreme gradient boosting (XGBoost), and tabular prior-data fitted network (TabPFN). These models represent distinct technical paradigms, i.e., neural network, instance-based learning, kernel method, tree-based ensemble learning, and foundation model, for tabular data (Figure 4). Among them, ANN establishes a mapping between input features and prediction targets through multilayer neurons and nonlinear activation functions. KNN generates predictions from the responses of neighboring samples in the feature space. SVR maps inputs into a high-dimensional space through a kernel function and performs nonlinear regression. Both RF and XGBoost are tree-based ensemble learning models, although they utilize different ensemble strategies. Based on bagging, RF combines the outputs of multiple independent decision trees to reduce the risk of overfitting. With boosting, XGBoost fits the residuals of preceding models to progressively improve overall prediction accuracy. TabPFN was pretrained on synthetic tabular tasks [31]. Through a transformer-based in-context learning mechanism, the model adopts the current training samples as prediction context to perform regression on new data. TabPFN version 7.1.1 was used in this study with a fixed configuration and without additional hyperparameter tuning.

#### 2.2.2. Model Training and Testing

Within a specific chloride concentration profile, input features related to concrete composition, environmental condition, and exposure duration were identical; only the exposed depth and corresponding chloride concentration varied. Therefore, a complete chloride concentration profile was utilized as the basic partitioning unit to ensure that data sampling points from one specific profile appeared exclusively in either the training or testing sets. The constructed dataset, composed of 701 chloride concentration profiles, was divided at an 8:2 ratio, i.e., a training set of 560 profiles and a testing set of 141 profiles. Before model training, numerical input features were standardized. The categorical exposure-zone variable was labeled as splash (0), tidal (1), and submerged (2) and subsequently transformed using one-hot encoding. For all six machine learning models, imputation statistics and feature scaling parameters were determined exclusively from the training data and then applied to the corresponding validation and testing data.

As mentioned, the number of observations in the splash, tidal, and submerged regions differed considerably. Therefore, the training and testing sets were stratified by exposure zone to maintain comparable proportions of the three exposure zones between the two subsets. For the training of ANN, KNN, SVR, RF, and XGBoost, Bayesian hyperparameter optimization was conducted with Optuna (version 3.1.0). Note that hyperparameter search was implemented exclusively within the training set. For each candidate hyperparameter configuration, the 560 profiles in the training set were randomly divided into five folds, employing the same partition strategy described above and making sure that no profile appeared simultaneously in the training and validation folds. Four folds were adopted to train the model and the remaining one for validation, and this process was repeated until all five training-validation runs were completed. The mean squared error (MSE) was calculated for each of the five validation folds, and their mean and variability were incorporated into the optimization objective, as expressed in Equation (2).

(2) $$J={\bar{\mathrm{MSE}}}_{\mathrm{val}}+\beta \sigma_{\mathrm{MSE},\mathrm{val}}$$

where ${\bar{\mathrm{MSE}}}_{\mathrm{val}}$ is the mean MSE of the five validation folds; *σ*_MSE,val_ represents the standard deviation of five validation MSEs; and β denotes the penalty coefficient, set to be 1.0 in the study to balance the mean validation error and its variability.

**Figure 5:**
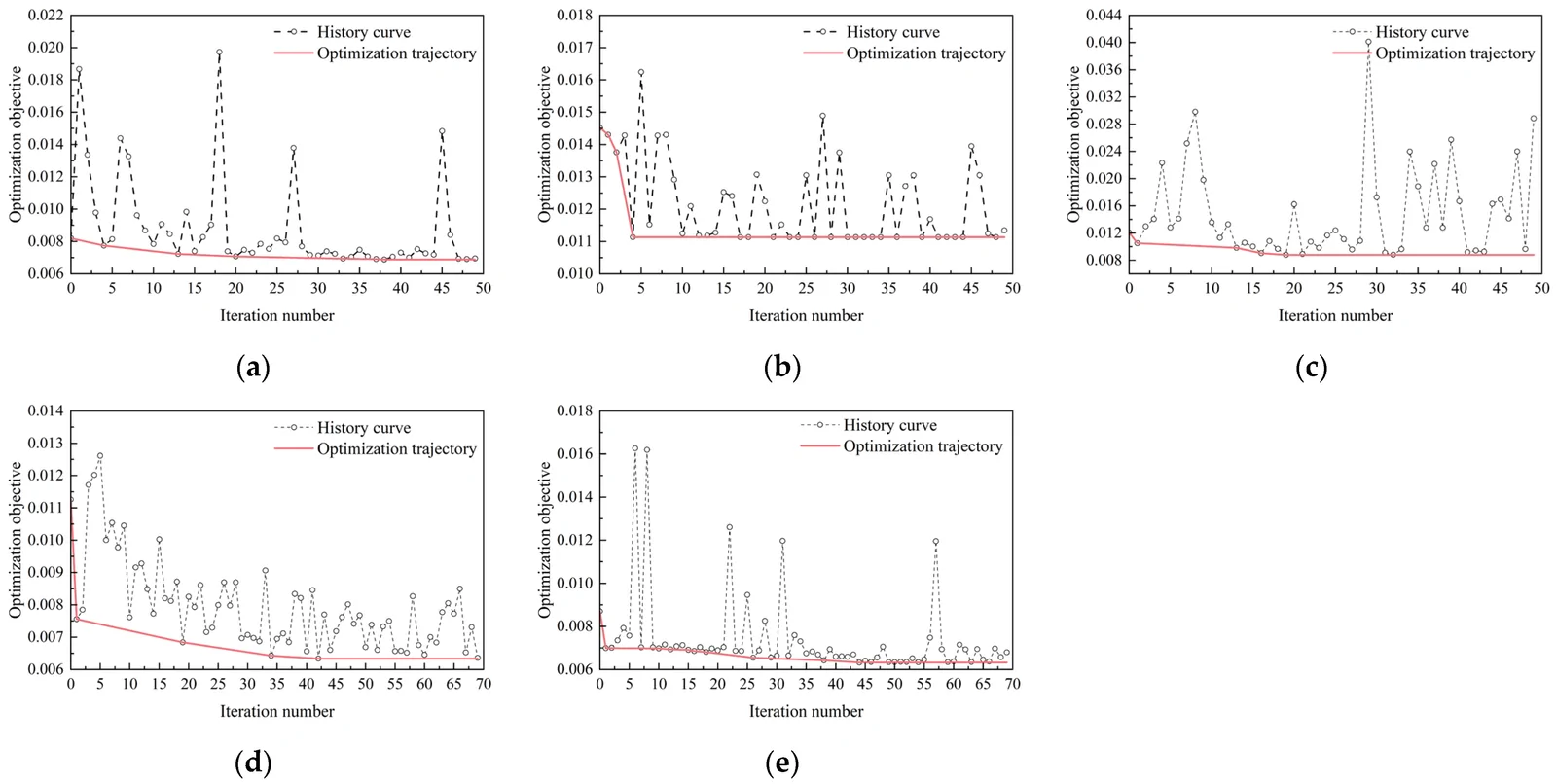
Bayesian hyperparameter optimization processes of different models: (**a**) ANN; (**b**) KNN; (**c**) SVR; (**d**) XGBoost; (**e**) RF.

The detailed hyperparameter search spaces and the number of Optuna trials of each model are provided in the Supplementary Materials. It should be noticed that TabPFN required no hyperparameter tuning. Figure 5 illustrates the Bayesian hyperparameter optimization processes for ANN, KNN, SVR, XGBoost, and RF. For each model, the best objective value was found to gradually decrease and stabilize as the number of trials increased, implying that the selected number of trials was sufficient for optimization. The final fixed hyperparameters of each model are provided in Table 2.

After the optimal hyperparameters were identified, ANN, KNN, SVR, RF, and XGBoost were retrained with the complete training set; as for TabPFN, the whole training set was utilized to establish the prediction context. The optimized hyperparameters were subsequently fixed across the 30 random-seed runs. All models were then evaluated on the corresponding testing set. To reduce the influence of data partitioning and model initialization on the evaluation results, dataset partitioning was repeated using 30 random seeds from 0 to 29. For each seed, the training and testing sets were repartitioned and the preprocessing procedure was repeated independently. This partitioning strategy was intended to evaluate the prediction performance on unseen chloride concentration profiles within the data domain represented by the compiled database and to assess the robustness of the evaluation results across different random data partitions and model initializations.

#### 2.2.3. Performance Evaluation

To evaluate the prediction performance of each model, two levels of performance metrics were adopted in this study: point-wise metrics and profile-level metrics. The point-wise metrics evaluated prediction performance over all individual measurement points, whereas the profile-level metrics assigned equal weight to each chloride concentration profile regardless of the number of measurement points it contains.

For the point-wise evaluation, the coefficient of determination (*R*^2^), root mean squared error (RMSE), and mean absolute error (MAE) were calculated as follows:

(3) $$R^{2}=1-\frac{\sum_{i=1}^{n}{\left(y_{i}-{\hat{y}}_{i}\right)}^{2}}{\sum_{i=1}^{n}{\left(y_{i}-{\bar{y}}_{i}\right)}^{2}}$$

(4) $$\mathrm{RMSE}=\sqrt{\frac{1}{n}\sum_{i=1}^{n}{\left(y_{i}-{\hat{y}}_{i}\right)}^{2}}$$

(5) $$\mathrm{MAE}=\frac{1}{n}\sum_{i=1}^{n}\left|y_{i}-{\hat{y}}_{i}\right|$$

where *y_i_* and ${\hat{y}}_{i}$ are the measured and predicted chloride concentrations for sample i, respectively; $\bar{y}$ is the mean of the measured values; and *n* is the number of samples.

For the profile-level evaluation, RMSE and MAE were first calculated separately for each chloride concentration profile and then averaged across all profiles. In this manner, each profile contributed equally to the overall performance assessment. The profile-level metrics were calculated using Equations (6) and (7). Profile-level *R*^2^ was not averaged across individual profiles because it is strongly dependent on the within-profile variance of the measured values. For profiles containing relatively few measurement points or limited variation in chloride concentration, individual-profile *R*^2^ may become unstable or undefined.

(6) $${\mathrm{RMSE}}_{\mathrm{profile}}=\frac{1}{K}\sum_{k=1}^{K}\sqrt{\frac{1}{n_{k}}\sum_{j=1}^{n_{k}}{\left(y_{\mathrm{kj}}-{\hat{y}}_{\mathrm{kj}}\right)}^{2}}$$

(7) $${\mathrm{MAE}}_{\mathrm{profile}}=\frac{1}{K}\sum_{k=1}^{K}\frac{1}{n_{k}}\sum_{j=1}^{n_{k}}\left|y_{\mathrm{kj}}-{\hat{y}}_{\mathrm{kj}}\right|$$

where *K* is the number of chloride concentration profiles, *n_k_* is the number of measurement points in the *k*-th profile, and *y_kj_* and ${\hat{y}}_{\mathrm{kj}}$ are the measured and predicted chloride concentrations at the *j*-th measurement point of the *k*-th profile, respectively.

## 3. Results

### 3.1. Model Performance

**Figure 6:**
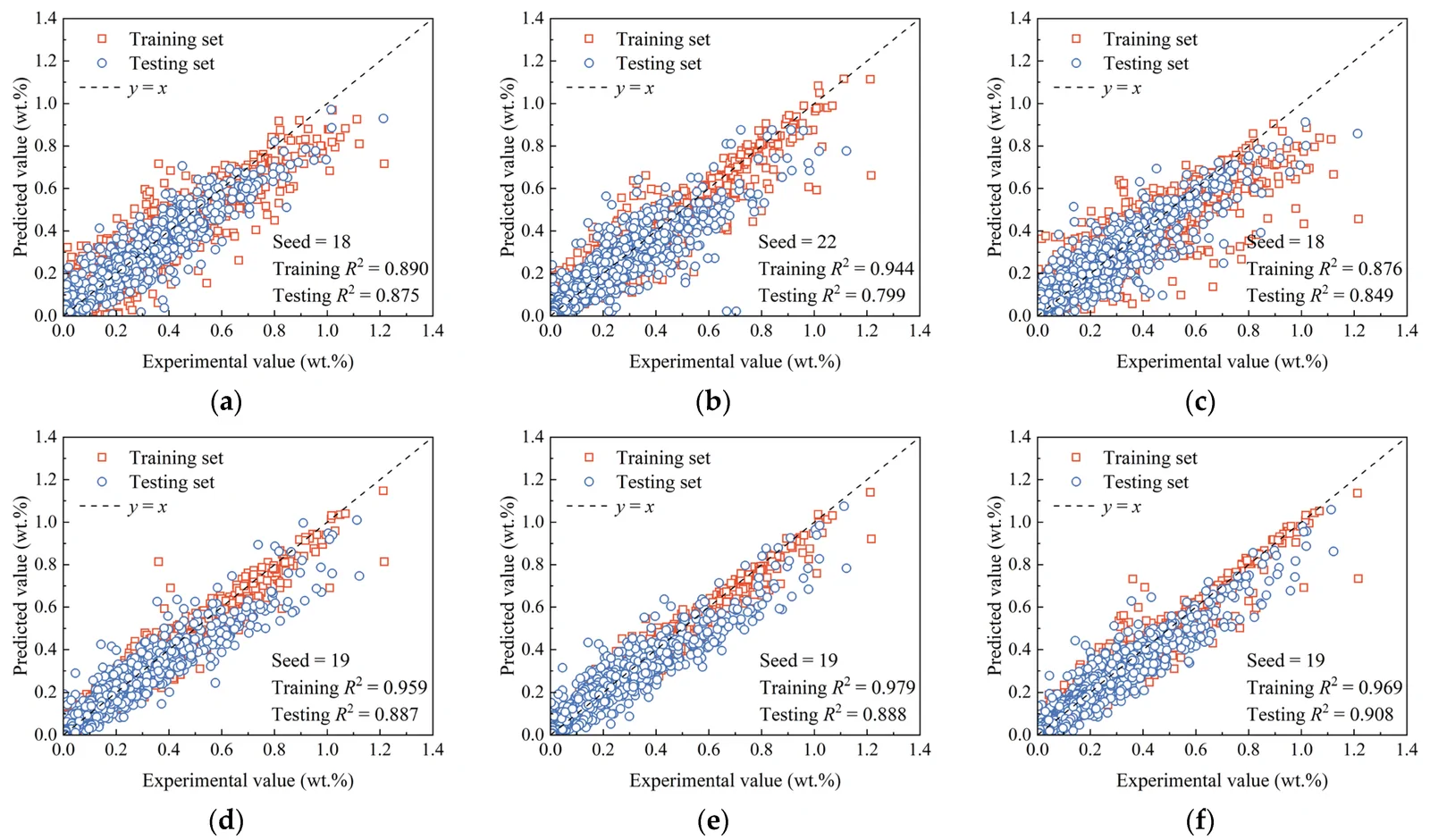
Comparison of predicted values from results of different models (run with the highest test-set *R*^2^) with experimental ones: (**a**) ANN; (**b**) KNN; (**c**) SVR; (**d**) XGBoost; (**e**) RF; (**f**) TabPFN.

Table 3 compares the overall prediction performance of the six models, with each metric reported as the mean ± standard deviation over 30 runs with different random seeds. For visualization, Figure 6 presents the predicted and experimental values from the run with the highest testing *R*^2^ for each model, and the overall model comparison based on the 30-run statistics is reported in Table 3.

As observed in Table 3, all models achieved relatively good performance under their corresponding best-seed run, with all testing *R*^2^ values close to or above 0.8. Among them, TabPFN yielded the best performance on the testing set. The mean *R*^2^ was 0.858, 0.067 for RMSE and 0.040 for MAE. Moreover, it is worth mentioning that the standard deviation of testing *R*^2^ of the model was only 0.025, implying that stable predictions were obtained across different random seeds. However, a single best result can be highly impacted by a specific data partitioning and may not be adequately representative. For example, KNN achieved a testing *R*^2^ of 0.799 with the best seed (seed = 22, see Figure 6), whereas the mean testing *R*^2^ across the 30 runs was 0.731 ± 0.052, with substantially higher variability than the others. It indicated that for datasets characterized by uneven distribution, evaluating a model from a single data partitioning can introduce notable bias. Comparing the mean and standard deviation across multiple random-seed runs was therefore recommended.

RF and XGBoost exhibited similar testing performance, with mean testing *R*^2^ values of 0.834 and 0.833, and a standard deviation of 0.022 for both. However, note that RF achieved the best training evaluation metrics (a mean training *R*^2^ of 0.982), and the difference between the mean training and testing *R*^2^ values was 0.149, suggesting overfitting to some extent. Similarly, the model showed lower training RMSE and MAE errors but comparatively higher testing errors. As for ANN, the difference between mean training and testing *R*^2^ values was only 0.085 (the smallest among these models), indicating a relatively balanced performance. On the other hand, despite a mean training *R*^2^ of 0.944, KNN produced the lowest mean testing *R*^2^ of 0.731. The difference between these two values reached 0.213, the largest among six models. In other words, the model showed evidence of overfitting and degraded generalization. In addition, KNN exhibited the largest standard deviation of testing *R*^2^, showing strong sensitivity to random data partitioning and local sample distribution.

In summary, TabPFN presented the best overall performance on the constructed dataset. RF and XGBoost followed, which also displayed good prediction stability. ANN and SVR illustrated relatively balanced training and testing performance, although their prediction accuracy was slightly lower. KNN gave a reduced generalization and presented pronounced sensitivity to data partitioning. Therefore, TabPFN was selected for the subsequent analysis based on its overall performance.

In addition to the point-wise evaluation, profile-level RMSE and MAE were calculated to assign equal weight to each chloride concentration profile, as described in Section 2.2.3. The corresponding results across the 30 random-seed runs are summarized in Table 4. The profile-level evaluation showed a generally consistent trend with the point-wise results. TabPFN again achieved the lowest testing errors, with a profile-level RMSE of 0.050 ± 0.0031 and MAE of 0.039 ± 0.0026. RF and XGBoost followed, with testing RMSE values of 0.056 ± 0.0031 and 0.056 ± 0.0032, respectively, and their corresponding MAE values were 0.045 ± 0.0028 and 0.046 ± 0.0029. KNN exhibited the largest testing errors at the profile level, with an RMSE of 0.073 ± 0.0049 and MAE of 0.059 ± 0.0047. These results confirmed that the overall comparison among the six models remained consistent when each chloride concentration profile was given equal weight. Note that the data distribution among exposure-zone groups was imbalanced, and the pooled and profile-level metrics should not be interpreted as evidence of uniform performance across exposure zones, durations, or material categories.

### 3.2. Model Interpretability

**Figure 7:**
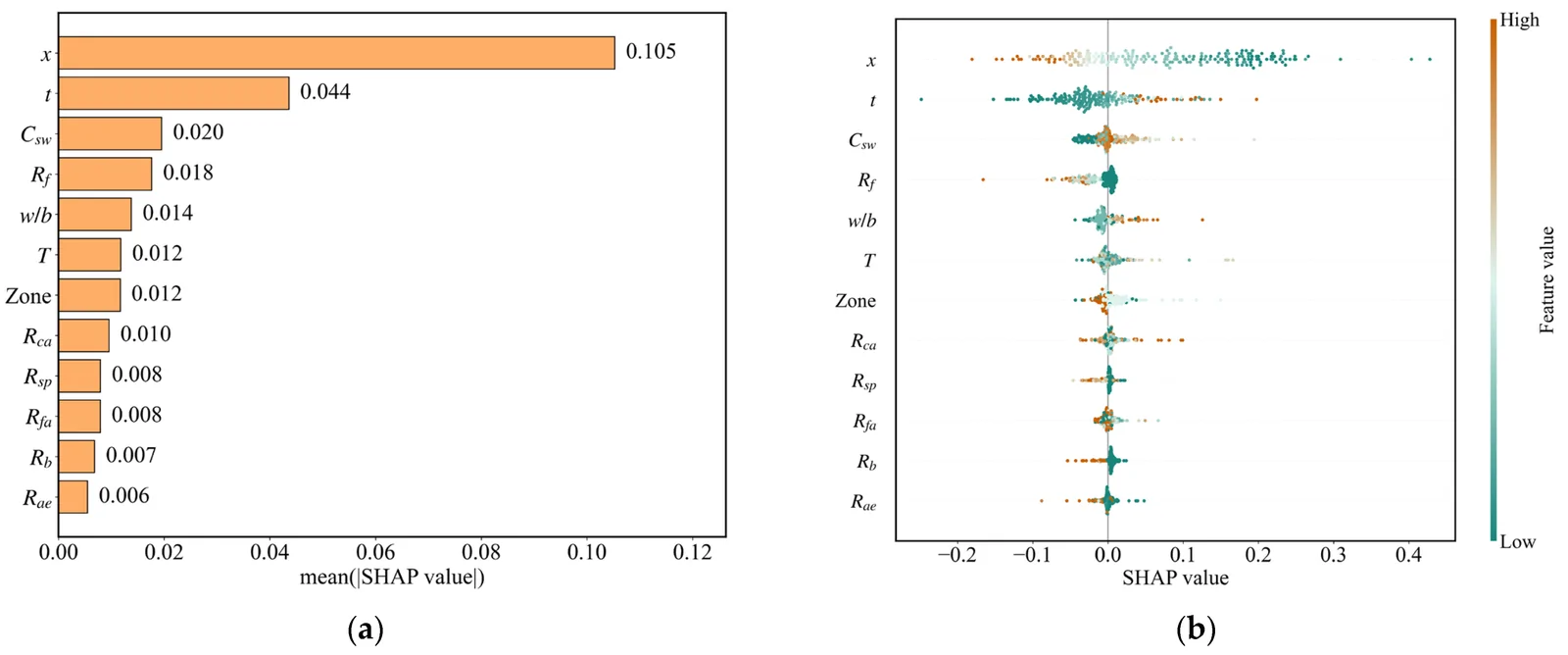
SHAP analysis results of the TabPFN model: (**a**) Mean absolute SHAP value of each feature; (**b**) beeswarm plot of SHAP values.

Although TabPFN achieved favorable mean predictive metrics on both training and testing sets, such models were generally regarded as “black box”, as their internal decision processes were difficult to explain. SHapley Additive exPlanations is a tool developed to address this issue. The central concept of SHAP originates from the Shapley value in cooperative game theory, which is used to distribute the benefits generated through cooperation among multiple participants and has been widely applied to interpretability analysis in recent years [32,33]. SHAP values were calculated using the SHAP PermutationExplainer. Figure 7 presents the SHAP analysis results of the TabPFN model. In Figure 7a, the mean absolute SHAP value of each feature, i.e., mean (|SHAP value|), is given, representing its global effect on model prediction. As can be seen, exposure depth x was the most important feature for predicting chloride concentration profile, with a mean absolute SHAP value of 0.105, markedly higher than the others. Exposure duration t was the next most influential feature, with a value of 0.044. These results indicated that exposure depth and duration constituted the principal features contributing to the model prediction. The remaining features, in descending order of importance, were *C_sw_*, *R_f_*, *w*/*b*, *T*, exposure zone, *R_ca_*, *R_sp_*, *R_fa_*, *R_b_*, and *R_ae_*. 

Beeswarm plot of SHAP values is depicted in Figure 7b. The horizontal axis represents the SHAP value: positive values indicate that features enhance the predicted chloride concentration, whereas negative values imply that they reduce the output. Point color denotes the magnitude of the feature, with warm colors representing higher values and cool colors for lower values. For exposure depth *x*, high-value samples were primarily distributed in the negative region, whereas low-value samples occurred more frequently in the positive part. In short, the predicted chloride concentration generally decreased with the increase in exposure depth. Conversely, high values of exposure duration t corresponded mainly to positive SHAP values and low values of exposure time for negative SHAP values. Therefore, longer exposure enhanced the content of chloride within concrete, reflecting the inward migration and internal accumulation of chloride over time.

Among the inputs of concrete composition, high values of *w*/*b* corresponded well to positive SHAP values, indicating that greater *w*/*b* was associated with higher predicted chloride concentration. It was reasonable, as a high *w*/*b* commonly generates a porous and interconnected microstructure, thereby facilitating chloride transport [34]. In contrast, high fly ash and slag contents were located primarily in the negative SHAP area, i.e., increasing SCM content in concrete can prohibit chloride ingress. This trend may be attributed to two mechanisms: (1) refinement of concrete pore structure through pozzolanic reactions between SCMs and portlandite, and (2) physical adsorption and chemical binding of chloride by their reaction products, e.g., C-S(A)-H gel and AFm phases [35]. On the other hand, the SHAP values of the coarse and fine aggregate features were generally small and dispersed across the horizontal axis, without a clear and stable monotonic trend. The addition of a water reducer and air entraining agent gave an overall negative trend in SHAP values, suggesting that appropriate admixture dosages may improve the resistance of concrete to chloride.

As for environmental condition variables, high values of *C_sw_* and *T* were noticed on the positive side. It was consistent with findings that high seawater chloride concentration and ambient temperature promoted chloride transport within concrete [36]. Subgroup analysis was categorically encoded as splash (0), tidal (1), and submerged (2). The beeswarm plot exhibited clear differences in SHAP values among these exposure zones. Tidal samples tended to provide positive contributions, whereas submerged samples made negative ones. It indicated that wetting–drying cycles in the tidal zone would intensify chloride absorption and diffusion, leading to a more severe chloride ingress [37].

Overall, the SHAP analysis confirmed that the interpretability of the TabPFN model was broadly consistent with well-known results of related research, providing an interpretable understanding of feature contribution to chloride concentration in concrete.

### 3.3. Parametric Investigation

To investigate the response of TabPFN under the selected parametric scenarios, it was adopted to parametrically predict chloride concentration profiles with various concrete compositions and environmental conditions. For all predicted profiles, the exposure depth ranged from 0 to 100 mm, with a sampling interval of 2 mm. The baseline parameters were specified in Table 5, and six representative features, i.e., *R_f_*, *w*/*b*, *R_sp_*, *T*, *C_sw_*, and *t*, covering three groups of impacting factors, were chosen for parametric analysis.

**Figure 8:**
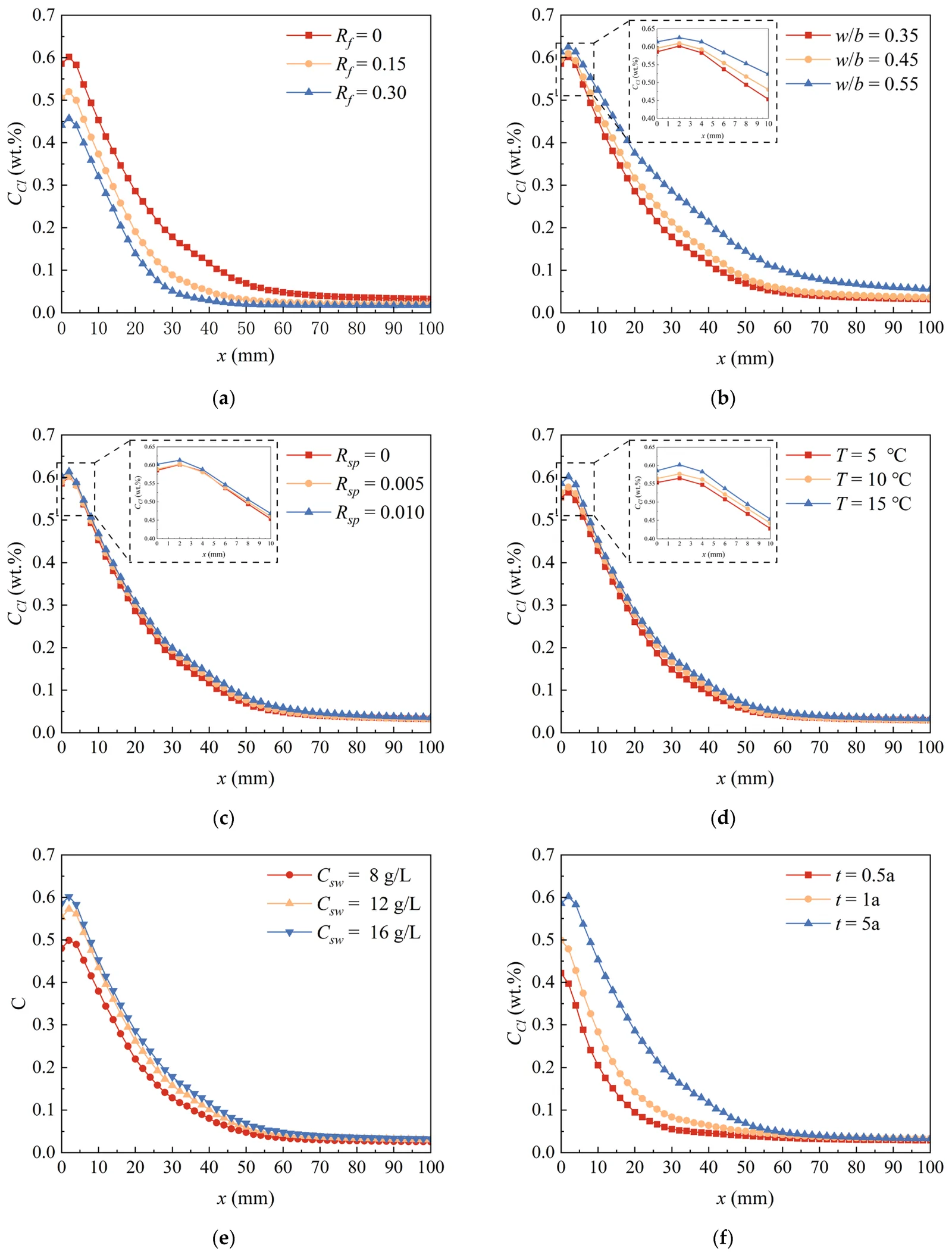
The predicted chloride concentration profiles of six representative features with varying input values: (**a**) fly ash proportion; (**b**) *w*/*b*; (**c**) water-reducer dosage; (**d**) mean ambient temperature; (**e**) seawater chloride concentration; (**f**) exposure duration.

Figure 8 illustrates the predicted chloride concentration profiles of six representative features with varying input values. As can be seen, all profiles exhibited the trend of decreasing chloride concentration with increasing exposure depth, together with a distinct concentration peak in the near-surface region. This pattern reflected the typical chloride distribution in concrete exposed to a tidal environment, where surface convection and internal diffusion acted simultaneously. In Figure 8a, increasing fly ash proportion from 0 to 0.30 reduced the overall chloride concentration and led to a more pronounced attenuation of chloride concentration with depth. Regarding the water-to-binder ratio (Figure 8b), enhanced *w*/*b* from 0.35 to 0.55 shifted the chloride concentration profile upward, with a rise in both the near-surface concentration and extent of chloride penetration. This behavior was sourced from the fact that a higher water-to-binder ratio increased the porosity and connectivity of concrete pore network, providing more favorable pathways for chloride transport. The profiles corresponding to different water reducer dosages were hard to distinguish, with only minor differences noted in the near-surface area (Figure 8c). It suggested that, within the investigated parameter range, the addition of a water reducer exerted a negligible effect on chloride transport.

With respect to environmental conditions, Figure 8d verifies that the rise of mean ambient temperature from 5 to 15 °C moved the chloride concentration profile upward slightly, as elevated temperature accelerated chloride transport. Additionally, raising the seawater chloride concentration from 8 to 16 g/L substantially intensified both the near-surface and internal chloride concentrations (Figure 8e). The effect of exposure duration is given in Figure 8f. It was found that both the chloride concentration and penetration depth progressively increased, and the profile extended further into concrete with the extension of exposure duration from 0.5 to 5 years.

Therefore, it can be concluded that within the investigated parameter ranges, the TabPFN model can reasonably describe the attenuation of chloride concentration with depth and effectively capture the influences of different factors on chloride diffusion within concrete. These parametric predictions agreed well with the established physical understanding of chloride transport in concrete, demonstrating that the model not only achieved satisfactory prediction performance but also learned the relationships among concrete composition, environmental condition, exposure duration, and chloride concentration.

### 3.4. Independent Prediction Case Study

In this section, the prediction performance of the TabPFN model was further examined using an independent experimental dataset [38], which was not contained in the constructed dataset. This study investigated the influence of sustained loading on chloride ingress in concrete, and the unloaded data was thus employed for validation. The specimens were exposed to the natural tidal environment at Xiaomai Island, Qingdao, China. The input values were determined according to the reported mixture design and environmental condition, i.e., *R_f_* = 0, *R_b_* = 0, *w*/*b* = 0.35, *R_fa_* = 1.5, *R_ca_* = 2.5, *R_sp_* = 0.002, *T* = 13.7°C, and *C_sw_* = 16 g/L.

**Figure 9:**
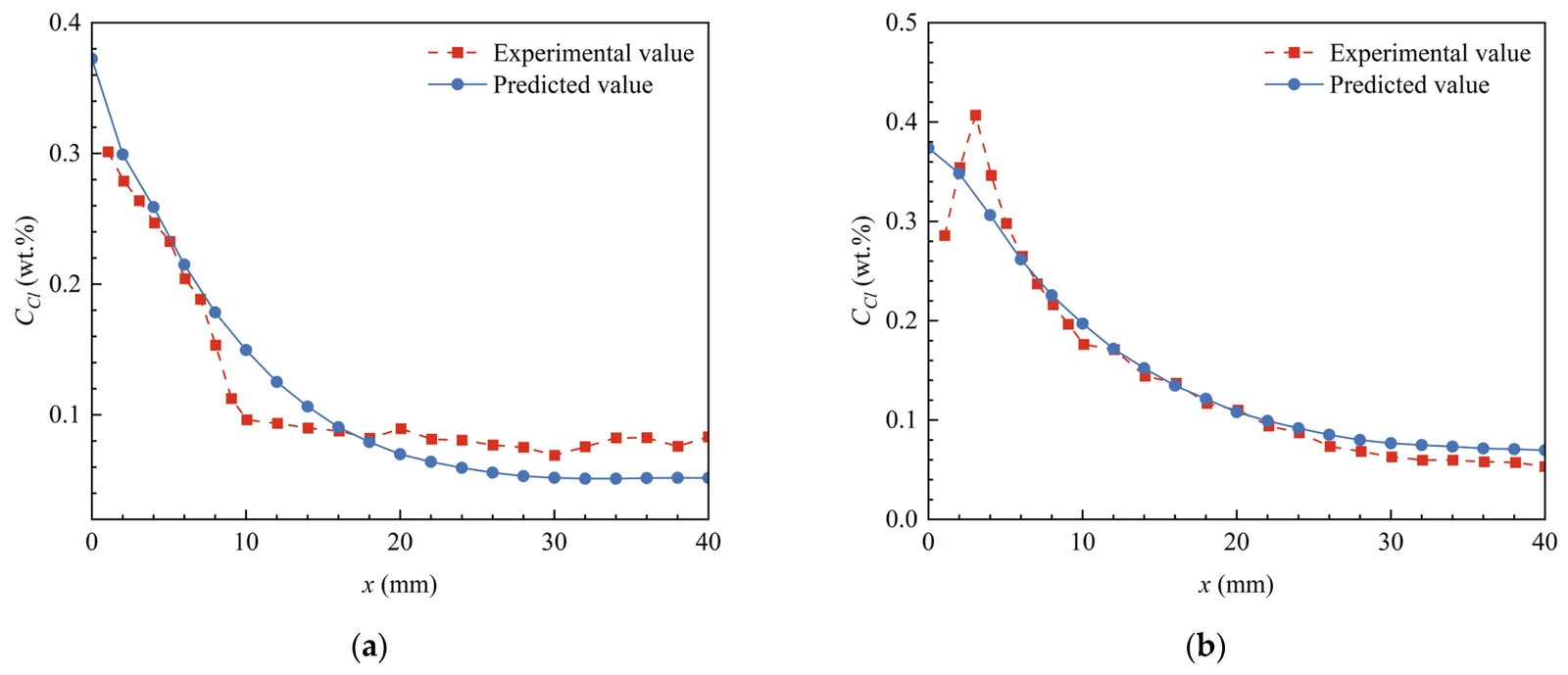
Comparison of predicted and experimental chloride concentration profiles: (**a**) 30 days; (**b**) 180 days.

Chloride concentration profiles after 30 and 180 days of exposure were predicted using the TabPFN model and compared with the experimental measurements, as shown in Figure 9. The predicted chloride concentration profile at 30 days agreed well with the experimental results, reproducing the overall decline of chloride concentration with exposure depth (Figure 9a). As for the 180-day exposure (Figure 9b), the experimental data showed a pronounced concentration peak in the near-surface convection zone, which was not fully captured by the model. Note that this discrepancy was mainly located in the near-surface region, and the predicted profile agreed closely with the experimental measurements over the remaining depth range. For durability assessment, chloride concentration at the reinforcement depth is of greater practical relevance than the near-surface peak.

It was also worth noting that this independent case study involved only one tidal environment, one mixture design, and two exposure durations. Therefore, it did not establish broader cross-environment generalization. Furthermore, the existing evaluation was designed to assess prediction across the pooled, heterogeneous database rather than to establish transferability to entirely unseen studies or sites. Further validation using data from independent studies and exposure sites is needed to evaluate the model’s generalization capability.

### 3.5. A GUI for Practical Applications

To facilitate the engineering applications of the TabPFN model, a graphical user interface was designed. The interface integrated model invocation, input preprocessing, chloride profile prediction, and data export within a unified platform, allowing users to obtain chloride concentration without accessing the underlying model code [39]. Considering that mixture design in engineering is generally expressed in terms of material mass, the GUI allowed users to directly enter the mass of each constituent. The corresponding input variables required by TabPFN were then automatically calculated.

**Figure 10:**
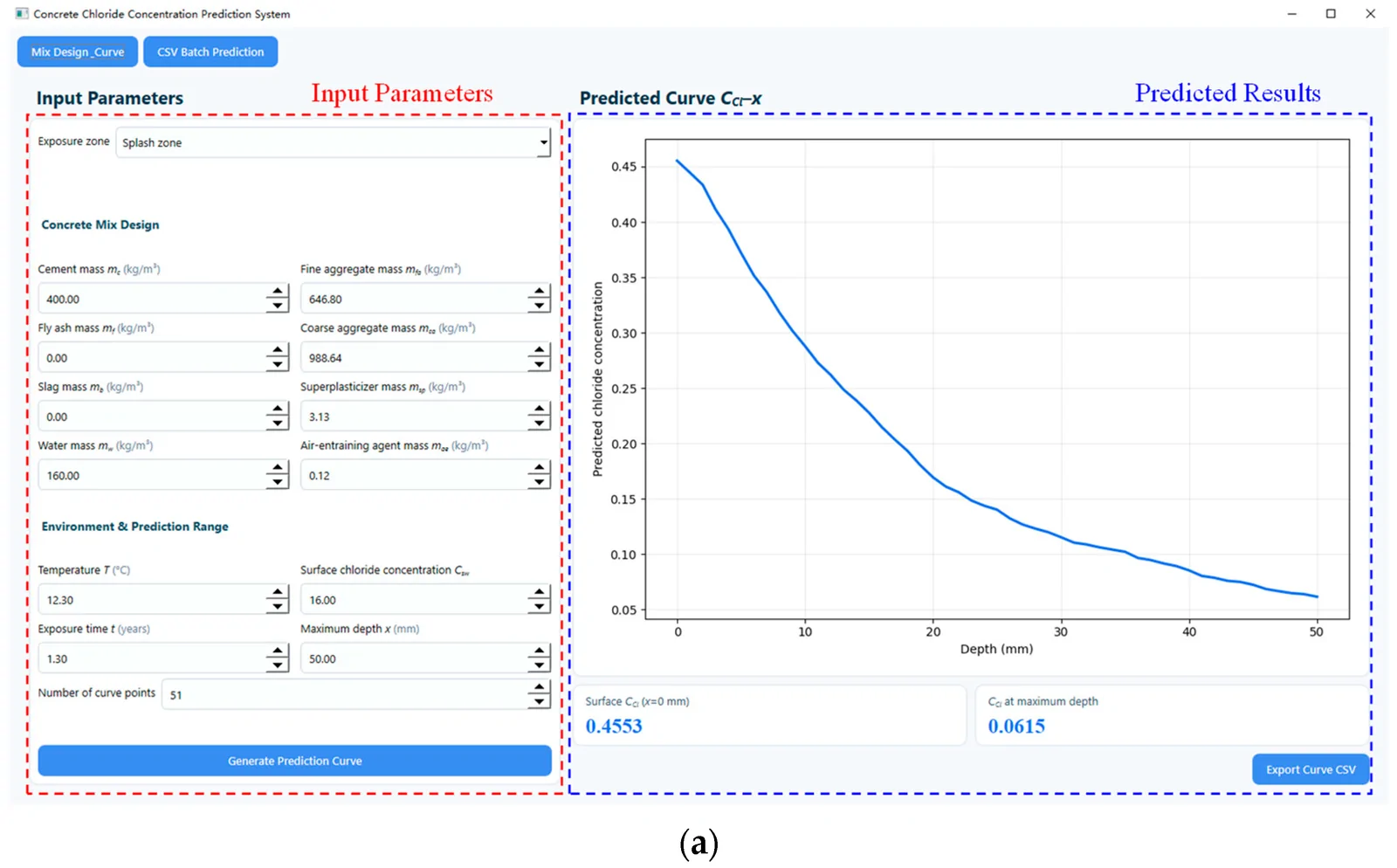

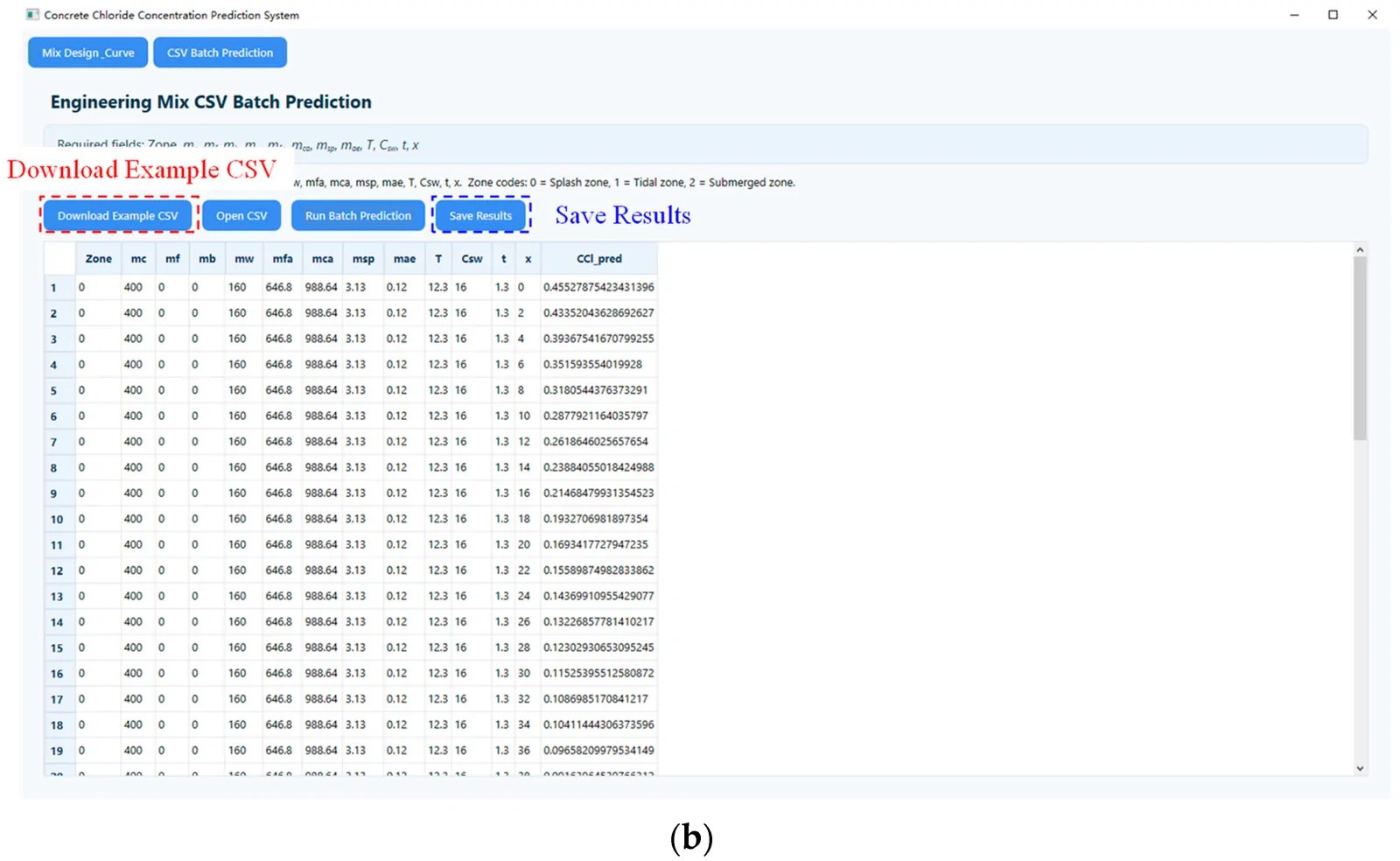
Graphical user interface based on the developed TabPFN model: (**a**) Single profile prediction; (**b**) CSV-based batch prediction.

The developed interface exhibited two main functions: single profile prediction and CSV-based batch prediction. For single profile prediction (Figure 10a), users specified the concrete composition, environmental condition, exposure duration, exposure zone, and range of prediction depth. With these input parameters, the corresponding chloride profile can be generated. The predicted chloride concentrations at the concrete surface and the specified maximum depth were also displayed, as well as the complete prediction data. The present interface provides deterministic, exploratory predictions; calibrated prediction intervals and a validated multivariate applicability-domain check are not currently available. Predictions for poorly represented input combinations should therefore be interpreted cautiously.

For engineering applications involving multiple concrete mixtures or exposure scenarios, a batch prediction module was incorporated (Figure 10b). Users can download a predefined CSV template and enter the values of variables for multiple cases. Once the CSV file is uploaded, the system conducts parallel predictions for all input cases. This function enabled efficient processing of large numbers of design alternatives and provided comparative evaluation and preliminary durability assessment of marine concretes.

## 4. Conclusions

The authors of this paper proposed an end-to-end machine learning method to predict the chloride concentration profile of concrete exposed to marine environments. A comprehensive multi-source chloride concentration dataset was constructed at first, followed by feature processing, hyperparameter optimization, and model training. Next, performance of six machine learning models was compared on both training and testing sets, and SHAP was adopted to examine the model interpretability. In addition, the potential of the best-performing TabPFN model for durability design was explored with parametric investigation and external validation. Finally, a GUI was designed to facilitate the engineering application of the proposed TabPFN model. The main conclusions are as follows:

1. Among these developed end-to-end prediction models, TabPFN performed best within the constructed dataset, achieving 0.858 ± 0.025 for testing *R*^2^, 0.067 for RMSE and 0.040 for MAE. On the other hand, despite a mean training *R*^2^ of 0.944, KNN produced the lowest mean testing *R*^2^ of 0.731.
2. SHAP analysis identified exposure depth and exposure duration as the dominant input features. It indicated that the spatial and temporal evolution of chloride concentration formed the principal basis for model prediction. The feature importance of the remaining variables decreased gradually, suggesting that inputs related to concrete composition and environmental condition contributed marginally to model prediction.
3. Through parametric investigation and external validation, it was found that the TabPFN model can reasonably describe the attenuation of chloride concentration with depth and effectively capture the influences of different factors on chloride diffusion within concrete. With the designed GUI, the proposed model can facilitate rapid chloride concentration profile estimation and provide useful insights for subsequent durability assessment. These observations are limited to the evaluated data domain and the specific independent case studies and do not establish calibrated prediction uncertainty or transferability to all marine exposure conditions.

Nevertheless, the present evaluation was primarily based on profile-level data partitioning, and the independent prediction was limited in scope. In particular, high-quality natural-exposure data involving different mixture designs remained scarce. When these data become available, more stringent data partitioning and broader external validation across different exposure conditions and longer exposure durations should be considered. In addition, no sensitivity analysis was performed to determine whether median imputation affected model ranking or interpretation. Future research should therefore evaluate the influence of different imputation strategies on model performance and interpretation.

## Figures and Tables

**Table 1:** Statistical summaries of 11 numerical feature variables and chloride concentrations.

Feature Variable	Symbol and Unit	Mean	Standard Deviation	Minimum	25th Percentile	Median	75th Percentile	Maximum
Fly ash-to-binder mass ratio	*R_f_*	0.093	0.142	0	0	0	0.178	0.50
Slag-to-binder mass ratio	*R_b_*	0.067	0.163	0	0	0	0	0.70
Water-to-binder ratio	*w*/*b*	0.429	0.080	0.30	0.360	0.40	0.480	0.883
Fine aggregate-to-binder mass ratio	*R_fa_*	1.693	0.345	1.162	1.50	1.617	2.0	3.608
Coarse aggregate-to-binder mass ratio	*R_ca_*	2.502	0.587	1.174	2.245	2.472	2.947	4.338
Water reducer-to-binder mass ratio	*R_sp_*	0.003	0.004	0	0	0	0.007	0.021
Air entraining agent-to-binder mass ratio	*R_ae_*	0.0005	0.0013	0	0	0	0.0003	0.0055
Mean ambient temperature	*T* (°C)	14.673	6.231	5.0	11.0	12.30	16.0	30.0
Seawater chloride concentration	*C_sw_* (g/L)	14.276	6.064	3.260	8.50	16.0	19.0	23.40
Exposure duration	*t* (year)	3.389	5.140	0.077	0.750	1.30	3.178	30.0
Exposure depth	*x* (mm)	18.356	18.004	0.374	6.033	14.037	25.0	152.823
Chloride concentration	*C_Cl_* (wt.%)	0.194	0.176	0	0.055	0.157	0.274	1.217

**Table 2:** The final fixed hyperparameters of each model.

Model	Hyperparameter	Optimal Value
ANN	Number of neurons in hidden layers	(128, 48)
	Activation function	ReLU
	L2 regularization coefficient	0.00021
	Initial learning rate	0.00047
	Batch size	32
	Dropout ratio	0.17848
KNN	Number of nearest neighbors	3
	Neighbor-weighting scheme	Uniform weighting
	Minkowski distance parameter	2
SVR	Kernel function	Radial basis function kernel
	Penalty parameter	0.61422
	ε-insensitive interval	0.00102
	Kernel coefficient	0.19262
XGBoost	Number of decision trees	1000
	Maximum tree depth	7
	Learning rate	0.01246
	Row-sampling ratio	0.92426
	Feature-sampling ratio	0.97003
	Minimum child weight	0.01994
	Minimum split loss	0.00226
	L1 regularization coefficient	0.18187
	L2 regularization coefficient	3.51451
RF	Number of decision trees	800
	Maximum tree depth	30
	Minimum sample required to split a node	2
	Minimum sample in a leaf node	1
	Feature-sampling ratio	0.75

**Table 3:** Point-wise prediction performance of different models across 30 random seeds.

Model	Training *R*^2^	Testing *R*^2^	Training RMSE	Testing RMSE	Training MAE	Testing MAE
ANN	0.895 ± 0.006	0.810 ± 0.035	0.057 ± 0.001	0.078 ± 0.007	0.039 ± 0.001	0.051 ± 0.004
KNN	0.944 ± 0.003	0.731 ± 0.052	0.041 ± 0.001	0.093 ± 0.009	0.024 ± 0.001	0.058 ± 0.005
SVR	0.889 ± 0.006	0.776 ± 0.039	0.058 ± 0.001	0.084 ± 0.006	0.032 ± 0.001	0.053 ± 0.004
XGBoost	0.964 ± 0.002	0.834 ± 0.022	0.033 ± 0.001	0.073 ± 0.004	0.023 ± 0.001	0.046 ± 0.003
RF	0.982 ± 0.001	0.833 ± 0.022	0.024 ± 0.001	0.073 ± 0.004	0.014 ± 0.001	0.046 ± 0.003
TabPFN	0.971 ± 0.003	0.858 ± 0.025	0.030 ± 0.002	0.067 ± 0.005	0.015 ± 0.001	0.040 ± 0.003

**Table 4:** Profile-level prediction performance of different models across 30 random seeds.

Model	Training RMSE	Testing RMSE	Training MAE	Testing MAE
ANN	0.050 ± 0.0012	0.064 ± 0.0037	0.041 ± 0.0012	0.052 ± 0.0033
KNN	0.036 ± 0.0006	0.073 ± 0.0049	0.027 ± 0.0004	0.059 ± 0.0047
SVR	0.045 ± 0.0008	0.066 ± 0.0040	0.032 ± 0.0006	0.053 ± 0.0035
XGBoost	0.028 ± 0.0004	0.056 ± 0.0032	0.023 ± 0.0004	0.046 ± 0.0029
RF	0.191 ± 0.0004	0.056 ± 0.0031	0.015 ± 0.0003	0.045 ± 0.0028
TabPFN	0.198 ± 0.0004	0.050 ± 0.0031	0.015 ± 0.0007	0.039 ± 0.0026

**Table 5:** Baseline parameters.

Baseline Parameters	Values	Baseline Parameters	Values
exposure zone	tidal zone	*R_sp_*	0
*R_f_*	0	*R_ae_*	0
*R_b_*	0	*T*	15 °C
*w*/*b*	0.35	*C_sw_*	16 g/L
*R_fa_*	1.6	*t*	5 years
*R_ca_*	2.4		

## Data Availability

The original contributions presented in this study are included in the article/Supplementary Materials. Further inquiries can be directed to the corresponding author.

## Supplementary Materials

The following supporting information can be downloaded at: https://www.mdpi.com/article/10.3390/ma19214509/s1. Refs. [6,18,28,40,41,42,43,44,45,46,47,48,49,50,51,52,53,54,55,56,57,58,59,60,61,62,63,64,65,66,67,68,69,70,71,72,73,74,75,76,77,78,79,80,81,82,83,84,85] can be found in Supplementary Materials.

## Abbreviations

The following abbreviations are used in this manuscript:

SHAP	SHapley Additive exPlanations
GUI	Graphical User Interface
SCMs	Supplementary Cementitious Materials
TabPFN	Tabular Prior-data Fitted Network
ANN	Artificial Neural Network
SVR	Support Vector Regression
KNN	K-nearest Neighbor
CNN	Convolutional Neural Network
LSTM	Long Short-Term Memory
OPC	Ordinary Portland Cement
RF	Random Forest
XGBoost	Extreme Gradient Boosting
*R*^2^	Coefficient of Determination
MSE	Mean Squared Error
RMSE	Root Mean Squared Error
MAE	Mean Absolute Error
